# A Custom Target Next-Generation Sequencing 70-Gene Panel and Replication Study to Identify Genetic Markers of Diabetic Kidney Disease

**DOI:** 10.3390/genes12121992

**Published:** 2021-12-15

**Authors:** Sonia Mota-Zamorano, Luz María González, Nicolás Roberto Robles, José Manuel Valdivielso, Bárbara Cancho, Juan López-Gómez, Guillermo Gervasini

**Affiliations:** 1 Department of Medical and Surgical Therapeutics, Institute of Molecular Pathology Biomarkers, Medical School, University of Extremadura, 06006 Badajoz, Spain; smotazamorano@gmail.com (S.M.-Z.); lmgonzalezgarcia89@gmail.com (L.M.G.); 2 Department of Nephrology, Badajoz University Hospital, 06006 Badajoz, Spain; nrrobles@unex.es (N.R.R.); bcancho@yahoo.es (B.C.); 3 Vascular and Renal Translational Research Group, Unidad de Prevención y Tratamiento de Enfermedades Cardiovasculares (UDETMA), Instituto de Salud Carlos III, REDinREN, IRBLleida, 25198 Lleida, Spain; valdivielso@irblleida.cat; 4 Service of Clinical Analyses, University Hospital, 06006 Badajoz, Spain; lopezhospi@yahoo.es

**Keywords:** chronic kidney disease, diabetes kidney disease, single nucleotide polymorphisms

## Abstract

Diabetic kidney disease (DKD) has been pointed out as a prominent cause of chronic and end-stage renal disease (ESRD). There is a genetic predisposition to DKD, although clinically relevant loci are yet to be identified. We utilized a custom target next-generation sequencing 70-gene panel to screen a discovery cohort of 150 controls, DKD and DKD-ESRD patients. Relevant SNPs for the susceptibility and clinical evolution of DKD were replicated in an independent validation cohort of 824 controls and patients. A network analysis aiming to assess the impact of variability along specific pathways was also conducted. Forty-eight SNPs displayed significantly different frequencies in the study groups. Of these, 28 with *p*-values lower than 0.01 were selected for replication. *MYH9* rs710181 was inversely associated with the risk of DKD (OR = 0.52 (0.28–0.97), *p* = 0.033), whilst *SOWAHB* rs13140552 and *CNDP1* rs4891564 were not carried by cases or controls, respectively (*p* = 0.044 and 0.023). In addition, the *RGMA* rs1969589 CC genotype was significantly correlated with lower albumin-to-creatinine ratios in the DKD patients (711.8 ± 113.0 vs. 1375.9 ± 474.1 mg/g for TC/TT; mean difference = 823.5 (84.46–1563.0); *p* = 0.030). No biological pathway stood out as more significantly affected by genetic variability. Our findings reveal new variants that could be useful as biomarkers of DKD onset and/or evolution.

## 1. Introduction

The total number of patients with chronic kidney disease (CKD) in the world has been estimated to be as high as 850 million. Among the pathologies leading to this condition, diabetic kidney disease (DKD), a serious kidney-related complication of type 1 and 2 diabetes that is present in up to 40% of diabetic individuals, has been pointed out as the most prominent [1]. In addition, DKD is also the leading cause of end-stage renal disease (ESRD), accounting for about half of incident dialysis patients in the United States. Given the rising global burden of diabetes, the incidence of DKD and therefore that of ESRD is bound to increase.

The facts that only a subset of diabetic patients develop DKD, that its prevalence varies across ethnicities and that DKD and ESRD both cluster in families strongly suggest an inherited genetic predisposition to this condition [2]. Accordingly, there has been a great number of candidate gene association studies in DKD, but unfortunately, to date, reported results have been mostly inconclusive [3]. The main reason for these inconsistencies is explained by the fact that there are still large knowledge gaps regarding the exact molecular mechanisms responsible for DKD [4], even though accumulating evidence indicates that podocyte loss, epithelial dysfunction and inflammation must play significant roles in DKD pathogenesis and progression.

Next-generation sequencing (NGS) is a massively parallel sequencing technology that offers ultra-high throughput, scalability and speed. This technique is becoming increasingly utilized in genetic association studies because it is less expensive and less time-consuming than conventional sequencing and allows for large numbers of reads. Indeed, this technique has been regarded as exceptionally promising for upgrading the management of kidney diseases [5]. It could help reduce therapeutic inertia, a prevalent problem in type 2 diabetes (T2D) that affects clinical outcomes [6], improve the quality of the therapy in subjects with youth-onset T2D, who may have been receiving treatment for decades [7], or even unveil reasons for the reported sex differences in DKD phenotypes [8]. To date, however, NGS has been barely used to assess the role of genetic variability in this disease.

In the present work, we have aimed to design a custom panel containing 70 candidate genes previously associated with DN in the literature. This panel was utilized to analyze samples in a discovery cohort with three groups: non-diabetic individuals, DKD patients and DKD patients with ESRD. An independent, larger validation cohort of cases and controls was later analyzed to confirm the relevance of the variants identified in the first stage.

## 2. Materials and Methods

### 2.1. Study Design

The first phase of the study consisted of a discovery cohort of 150 Caucasian subjects recruited from three different hospitals in Badajoz (Spain) over a 30-month period (June 2017 to December 2019). These 150 individuals were grouped into three cohorts of 50 participants each, namely non-diabetic individuals, DKD patients and DKD patients who, at the time of the study, had ESRD and were on dialysis. Participants in the three groups were matched by sex and age. In addition, selected diabetic patients on dialysis had a similar duration of T2D as compared with the DKD patients.

A 10 mL blood sample was collected at the time of the participants’ visit to the hospital and stored at −80 °C until analysis. All participants had to be over 18 years of age to be included in the study. DKD patients all had T2D prior to the presence of kidney disease, defined as albuminuria or estimated glomerular filtration rate (eGFR) <60 mL/min. According to the American Diabetes Association diagnostic criteria, T2D was defined as fasting glucose ≥126 mg/dL (7.0 mmol/L) or a 2-hour plasma glucose level ≥200 mg/dL (11.1 mmol/L) during a glucose tolerance test with an oral administration of 75 g. DKD was diagnosed by biopsy (performed when proteinuria was greater than 1 g/day) and/or by clinical criteria (presence of both retinopathy and albuminuria after excluding other possible causes).

In the second phase of the study, an independent validation cohort was analyzed to test the association of the SNPs whose frequencies were significantly different between the three groups of the discovery cohort. DNA samples of 506 controls and 318 DKD Caucasian patients were obtained from the repository created by the NEFRONA project, an observational, prospective and multicenter study of cardiovascular morbidity and mortality in patients at different stages of CKD distributed throughout the Spanish territory [9]. Diagnosis criteria for DKD were the same as those used in the discovery cohort.

Written informed consent was obtained from all the participants. The study protocol was approved by the Ethics Committee of the Badajoz University Hospital and was carried out in accordance with the Declaration of Helsinki and its subsequent revisions.

### 2.2. Targeted Next-Generation Sequencing

DNA purification from blood samples was conducted with a standard phenol–chloroform extraction and ethanol precipitation. DNA samples were then stored at 4 °C in sterile plastic vials. In the case of participants recruited in the NEFRONA study, genetic material was obtained from biological samples stored at the REDinREN biobank [10] using QIAamp DNA Blood Kits (Qiagen, Hilden, Germany).

Targeted NGS was applied to a custom-made panel of 70 genes in the discovery cohort. These genes were selected using online queries of GeneCards (www.genecards.org; accessed on 14 July 2020), Ensemble (www.ensemble.org; accessed on 11 July 2020), Online Mendelian Inheritance in Man (OMIM; www.omim.org; accessed on 10 July 2020) and a thorough review of the literature that identified reports of their putative involvement in DKD. We also included a group of 16 genes whose participation in DN has been suggested by different GWAS [8]. The DNA corresponding to the 150 participants in this phase was purified from whole blood samples using a standard phenol–chloroform extraction method. The NGS panel was designed with the Ion AmpliSeq On-Demand Panels for targeted sequencing (Thermo Fisher Scientific, Waltham, MA, USA). We used the Ion AmpliSeq Designer tool (Thermo Fisher Scientific, Waltham, MA, USA) to construct oligonucleotide pairs that covered the sequence of the 70 candidate genes (coding and regulatory regions as well as intron–exon boundaries). These oligos generated a total of 1527 amplicons offering a 97.2% coverage of the regions of interest. The oligos were divided into 2 pools to carry out the amplifications. The total sequenced area spanned 264 kb, consisting of 970 exons plus the 5′ and 3′ regulatory areas of each gene.

Sequencing was carried out by the Ion Torrent method in an Ion S5-Xl sequencer (Thermo Fisher Scientific, Waltham, MA, USA), which measures changes in pH resulting from hydrogen ions released by the addition of dNTPs to DNA polymers. The chosen NGS approach introduces a PCR-based sequence enrichment step using Ion AmpliSeq technology that targets genes of interest. The sequencer utilizes the Ion 530 Chip Kit-4 Reactions, which processes 16 samples for a total of 24,432 amplicons and generates up to 12 million reads, thus resulting in an estimated 500× coverage for each amplicon.

### 2.3. Bioinformatic Analyses

First, a quality control of the reads in FASTQ format that were generated by the sequencer was performed with the FastQC tool v0.11.4 (https://www.bioinformatics.babraham.ac.uk/projects/fastqc/; accessed on 12 October 2020). Next, in order to obtain a good alignment of the sequences, the adapters generated by the creation of the libraries, primers, poly-A tails and other unwanted sequences were removed with Cutadapt v1.13 (https://cutadapt.readthedocs.io/en/stable/; accessed on 12 October 2020). Reads were aligned against the sequence of the GRCH37 genome assembly using the Burrows–Wheeler Aligner (BWA-MEM 0.7.15-r1140, http://bio-bwa.sourceforge.net/bwa.shtml; accessed on 12 October 2020), which uses an algorithm to map slightly divergent sequences against a large reference genome. The output files (in SAM format) were then processed to eliminate duplicate readings, and the sequences were ordered to obtain a BAM format file. Then, VCF files including all the variants found were generated with BCFtools v1.6 (http://samtools.github.io/bcftools/bcftools.html; accessed on 12 October 2020). Finally, the variants contained in the VCF files were annotated with a number of human databases using the ANNOVAR tool (http://annovar.openbioinformatics.org; accessed on 12 October 2020).

### 2.4. Selection of Variants

DKD patients and controls were compared to identify genetic variants displaying significantly different frequencies. In addition, we also compared DKD patients with and without ESRD sharing a similar duration of diabetes to identify additional relevant SNPs for the disease. Finally, we also identified SNPs that displayed a significant effect on the eGFR and proteinuria values of the DKD patients. Variants were selected for subsequent replication analysis when they had a *p*-value < 0.01 in the aforementioned analyses and a MAF <0.05 in any of the comparison groups (to avoid the inclusion of frequent variants that were potentially less clinically relevant).

### 2.5. Gene–Gene Interaction Analysis

Interactions between the 70 candidate genes were evaluated with the GeneMANIA plugin in Cytoscape [11]. The genes were added to the query box of the search engine and the max resultant genes function (inclusion of other related genes) was set to zero. Network weighting was created based on the Gene Ontology (GO) biological processes database (www.geneontology.org, accessed on 12 June 2021). GeneMANIA assigns weights by linear regression to maximize connectivity between all input genes. Each network data source is represented as a weighted interaction network where each pair of genes is assigned an association weight; the higher the weight, the thicker the link appears in the figure. Two genes appear linked when there are data on co-expression, physical interaction or co-localization; there are predicted functional relationships; they participate in the same reaction within a pathway; they share protein domains; or if the effects of perturbing one gene have been found to be modified by perturbations to a second gene. A list of databases from where these data are retrieved can be found in www.genemania.org (accessed on 12 June 2021).

### 2.6. In Silico Study

Four different bioinformatics tools were utilized to predict functional effects of the SNPs that were found to be relevant both in the discovery and validation cohorts. These included: SIFT—Sorting Intolerant From Tolerant [http://sift.jcvi.org/www/SIFT_seq_submit2.html; accessed on 29 July 2021], PROVEAN—Protein Variation Effect Analyzer [http://provean.jcvi.org/index.php; accessed on 29 July 2021], PolyPhen-2—PolymorphismPhenotyping v2 [http://genetics.bwh.harvard.edu/pph2; accessed on 29 July 2021] and SNPs&GO [http://snps.biofold.org/snps-and-go/snps-and-go.html; accessed on 29 July 2021].

### 2.7. Statistical Analysis

Quantitative variables are shown as median and interquartile range values, and their comparison was carried out with Mann–Whitney/Student’s t-test or Kruskal–Wallis/ANOVA tests, depending on the normality of the data and the number of groups. Chi-square or Fisher’s tests were performed to detect differences between categorical variables and the frequency of the identified variants across the different cohorts. Data on proteinuria and estimated glomerular filtration rate (eGFR) were converted to binary variables, and sex- and age-adjusted logistic regression models were then used to assess the association between these parameters and the considered SNPs. Statistical analyses were performed using the SNPassoc package in the R environment.

## 3. Results

### 3.1. Discovery Cohorts

Table 1 shows the demographic and clinical characteristics of the three study groups, namely controls and DKD patients with and without ESRD. Age and sex were similar across the three groups (*p* > 0.05). Statistically significant differences were observed between the three groups for the incidence of smoking, hypertension and hyperlipidemia (*p* < 0.05). The duration of the disease was not significantly different between DKD patients with and without ESRD (*p* > 0.05; Table 1).

**Figure 1:**
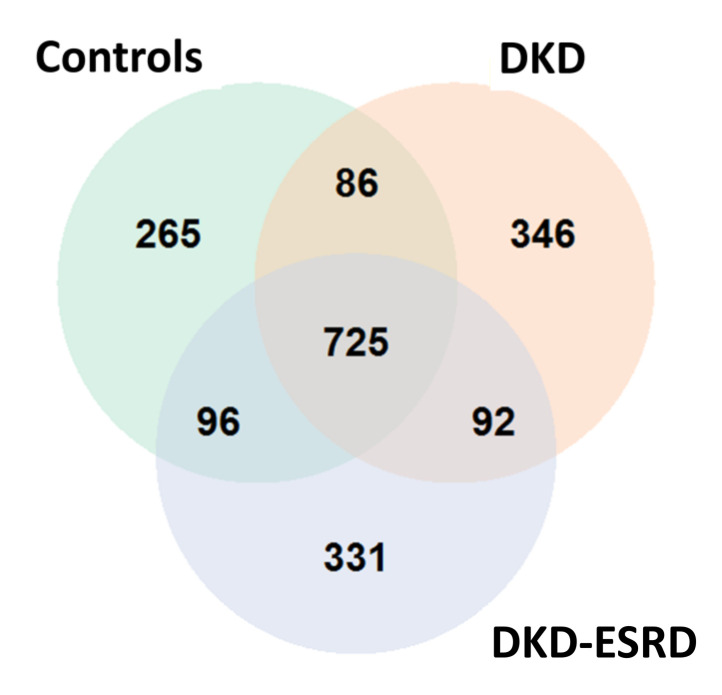
Venn diagram showing the distribution of identified genetic variants in controls and DKD patients with and without end-stage renal disease (ESRD).

A total of 1941 genetic variants were detected in the study population. Distribution of the variants in the three cohorts is depicted in Figure 1.

**Figure 2:**
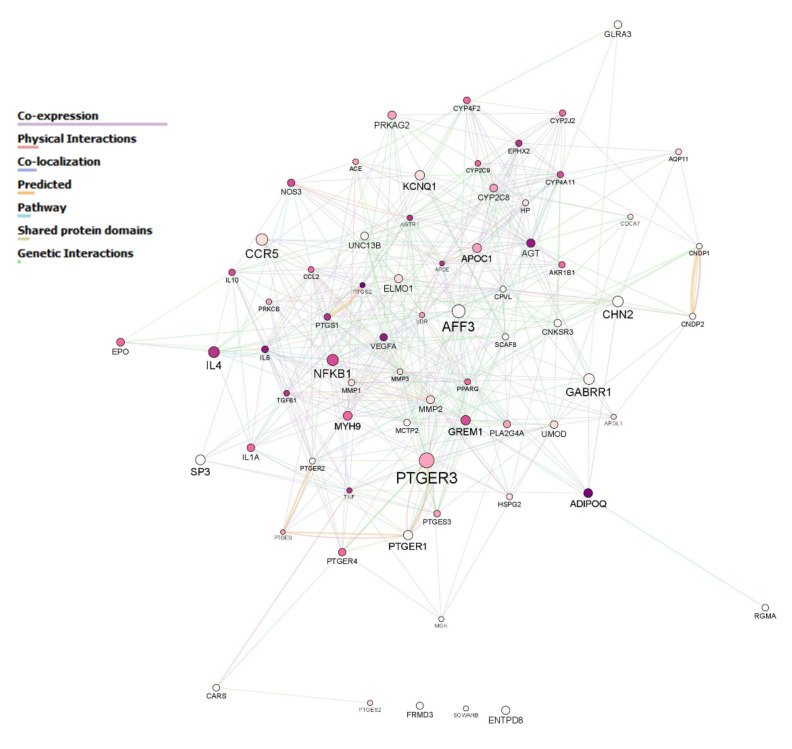
Seventy candidate genes included in the study. Each node corresponds to a gene. Their sizes are related to the number of variants harbored in the gene locus identified in the entire population. The more variants, the larger the size. The color of the node corresponds to the number of major biological pathways in which the gene participates; the darker the color, the more pathways are involved. White nodes represent genes that did not participate in any of the most common biological routes observed for the 70 genes according to the GO annotation for biological processes. The different color-coded interactions are specified in the inset.

**Figure 3:**
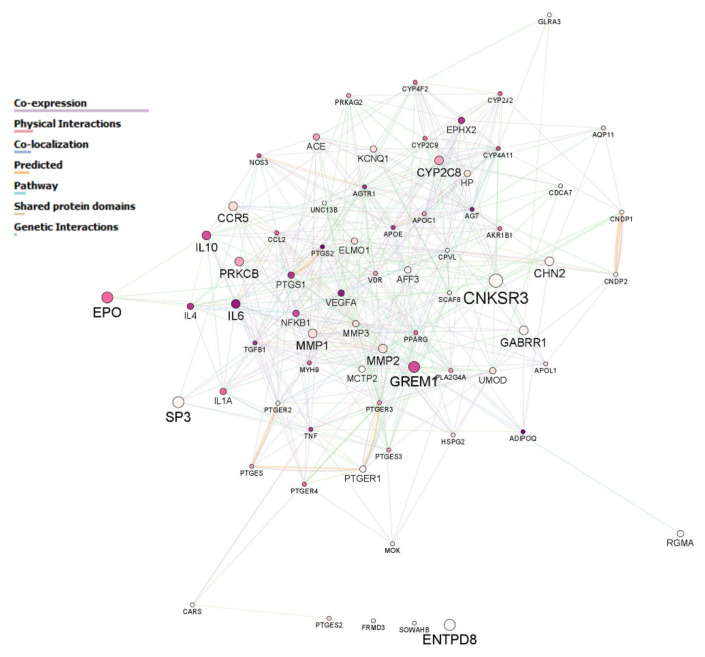
Candidate genes included in the study. The size of each node (gene) corresponds with the number of variants harbored in the gene locus, whose frequencies were significantly different between controls and patients. The more variants, the larger the size. The color of the node corresponds to the number of major biological pathways in which the gene participates; the darker the color, the more pathways are involved. White nodes represent genes that did not participate in any of the most common biological routes observed for the 70 genes according to the GO annotation for biological processes. The different color-coded interactions are specified in the inset.

Figure 2 shows the 70 candidate genes included in the panel and their biological interactions. The gene that presented a higher degree of variability in the population of study was *PTGER3* followed by *AFF3* and *CCR5*. The least polymorphic genes were *PTGES* and APOE, which harbored less than 10 variants each for the entire population. Figure 3 depicts the network of genes with node sizes according to the number of variants with a *p*-value < 0.05 in the comparison between controls and DKD patients. *CNKSR3* had four of these relevant SNPs, whilst *GREM1*, *EPO*, *ENTPD8* and *SP3* had three relevant variants each (Figure 3). The complete set of SNPs achieving a significant *p*-value is listed in Supplementary Table S1.

According to the GO annotation, the most common biological pathways in which the 70 candidate genes were involved were lipid metabolic process, inflammatory response, cell migration, circulatory system process, cell differentiation, cell death, arachidonic acid metabolism, steroid metabolic process, renal system development, response to oxidative stress and angiogenesis. ADIPOQ and PTGS2 were the genes involved in more pathways (seven). Twenty genes had no GO annotation or did not participate in the most frequent pathways observed. Supplementary Figure S1 shows the number of variants detected for each cohort in the 11 main biological routes involved. No statistically significant differences between the study groups were found for any of the pathways.

Table 2 shows the list of 28 gene variants found that displayed relevant associations in the discovery cohorts (*p*-values < 0.01) and were therefore subsequently subjected to validation in an independent study group. Six of these SNPs could not be included in the array design for technical reasons and hence we had to include six variants with *p*-values between 0.01 and 0.05 instead. Twenty-two out of the 28 variants were significantly associated with the risk of DKD, with *p*-values for the association ranging from 3.82 × 10^−6^ to 0.04. Most notably, a T>A substitution in the 77300435 position of the AQP11 gene (no rs number assigned) showed more consistent association with increased DKD risk (OR = 4.33 (2.21–8.57; *p* = 3.82 × 10^−6^). Two other SNPs, rs13140552 and rs41380244, displayed higher OR values of 11.00 and 12.23, respectively, but confidence intervals were very wide because of the low allele frequency in the limited size of the discovery cohort. In contrast, several other variants displayed a solid association with lower susceptibility to DKD (Table 2). Two additional SNPs were inversely associated with the onset of ESRD, namely rs470558 (OR = 0.15 (0.03–0.69), *p* = 0.010) and rs1969589 (OR = 0.08 (0.01–0.64), *p* = 0.005). Finally, four variants were associated with a higher risk of worse renal function or damage, as indicated by eGFR and proteinuria values with *p*-values for the association ranging from 0.004 to 0.026 (Table 2).

### 3.2. Validation Study

Information on the clinical and demographic parameters of the 824 controls and DKD patients included in the replication cohort is shown in Table 3. The control group was younger on average (*p* < 0.01) and included a higher percentage of females (*p* < 0.01) than the group of DKD patients. Statistically significant differences were also seen for hypertension and hyperlipidemia (*p* < 0.00001; Table 3).

Of the total of 28 SNPs studied in the replication cohort, three of them, namely rs13140552 in the *SOWAHB* (also known as *ANKRD56*) gene, rs4891564 located in the 3′UTR region of *CNDP1* and rs710181 in *MYH9*, still displayed a significant influence on the risk of DKD, as calculated by logistic regression adjusting for sex and age. Table 4 shows genotype distributions between controls and patients. Odds ratio (OR) values for rs13140552 and rs4891564 could not be calculated, as the variant was not present in one of the study groups. *MYH9* rs710181 was found to be inversely associated with the risk of DKD (OR = 0.52 (0.28–0.97), *p* = 0.033; Table 4). In addition, one last SNP, *RGMA* rs1969589 T/C, significantly correlated with lower proteinuria values. Carriers of the homozygous CC genotype had albumin-to-creatinine ratios of 711.8 ± 113.0 mg/g vs. 1375.9 ± 474.1 mg/g for TC/TT carriers (mean difference = 823.5 (84.46–1563.0); *p* = 0.030).

Only two of these four relevant SNPs were recognized by the function predicting tools utilized (SIFT, PROVEAN, SNPs&GO and PolyPhen-2). These were rs13140552 (Pro291Ser) and rs710181 (Ala1143Ala), which were both tagged as Tolerated/Neutral/Benign.

## 4. Discussion

Genetic testing has been proved useful in a variety of heritable kidney diseases; however, its application in DKD, with the exception of some studies on African American populations [12,13], has been very limited. In this work, we conducted a massive parallel sequencing of 70 candidate genes for DKD and replicated significant results in an independent cohort in order to identify genetic biomarkers of the disease.

The results of our replication study confirm that three genetic variants identified in the discovery cohort, namely rs13140552, rs4891564 and rs710181, could constitute susceptibility loci for DKD. Of these, rs710181 in the *MYH9* gene was found to be carried by a relatively significant percentage of the study population (8.1 and 4.7% of controls and cases, respectively). The non-muscle myosin heavy chain IIA protein encoded by *MHY9* is highly expressed in podocytes. Precisely, injury to podocytes leading to proteinuria and glomerulosclerosis is considered to be a major contributor to DKD [14]. Indeed, Kang et al. recently revealed the mechanism linking *MHY9* to DKD, demonstrating that an angiotensin II-mediated *MHY9* downregulation causes structural and functional podocyte injury, thus increasing filtration barrier permeability [15]. Moreover, genetic variability in the gene locus has also been shown to alter podocyte structure, making these cells more injury-prone after a damaging stimulus [16], and *MYH9* SNPs have been shown to be associated with ESRD linked to diabetes in Europeans and African Americans [17,18]. This background supports the findings presented herein on the putative clinical relevance of *MHY9* rs710181 SNP for DKD. It should be remarked that rs710181 causes a C-to-A substitution in exon 26 of the gene locus, which translates into a synonymous Ala1143Ala polymorphism. The fact that a synonymous variant may have clinical consequences is not as uncommon as one might think. There are many examples in the literature illustrating that synonymous polymorphisms should not automatically be disregarded in genomic analyses. A number of studies have pointed out that synonymous mutations may affect translation kinetics, miRNA binding, splicing machinery or mRNA stability and lead to altered protein function [19,20]. In this regard, even though cancer has been the field where this type of mutation has been mostly studied, there is also evidence that synonymous SNPs may contribute to renal pathologies such as nephronophtisis [21] or polycystic kidney disease [22,23]. Interestingly, this synonymous rs710181 SNP in particular was selected for analysis in an association study with glomerulosclerosis in African Americans; however, its effect could not be tested, as the frequency shown in these ethnicity was too low [24]. In any case, rs710181 has been pointed out as a tag-SNP of the *MYH9* gene locus [25], e.g., a representative variant of an haplotype block. Therefore, we should not rule out that the observed effect of rs710181 could in fact be produced by another SNP in high linkage disequilibrium with this variant.

With regard to the other two SNPs that were pinpointed in the validation study, namely rs13140552 and rs4891564, the latter, in *CNDP1*, had previously been found to be related to DKD risk in African Americans [26]. Our findings would therefore confirm this association in Caucasian patients as well. *CNDP1* encodes the enzyme responsible for the degradation of carnosine, a peptide with renoprotective properties. This gene has been pointed out as an important locus for DKD [27], as its overexpression, enhancing carnosine metabolism, may favor the development of this complication [28]. The identified rs4891564 SNP is located in the regulatory region of the gene and hence it holds the potential to affect its expression. Much less data is available on the other relevant SNP, *SOWAHB* rs13140552. One report that analyzed DKD biopsies linked downregulation of *SOWAHB* expression to the disease, but the specific role of this gene in the context of DKD remains to be assessed [29]. In any case, it should be acknowledged that these two SNPs were carried by very few patients and hence caution should be exerted when interpreting these results.

Finally, the C-allele of the *RGMA* rs1969589 SNP was observed to play a protective role in the discovery cohort, as it resulted in an inverse association with diabetic ESRD onset, a finding that has also been reported for other *RGMA* variants [30]. This association with ESRD was, however, not maintained in the validation study, but a significant correlation with lower proteinuria values was found instead, which could explain this putative protective role. Given that the rs1969589 SNP has been proposed to alter the binding of microRNAs [31], it seems likely that the mechanism underlying the observed effects was the modulation of gene expression.

In this work we also aimed to study the influence of genetics in pathways that are proposed to be involved in DKD pathogenesis. However, we could not confirm that any of these biological routes in which the 70 candidate genes participate displayed a significantly different number of genetic variants between controls and DKD patients. Therefore, and at least from the point of view of our genetic approach, no pathway was observed to play a predominant role in the susceptibility for the disease. This is most likely a reflection of the high complexity and extremely multifactorial character of the pathogenic mechanisms of DKD, which are not fully understood yet [32].

This study has several limitations. We aimed to identify variants that are not common in the study population (Caucasian Spaniards) and hence have a higher likelihood of clinical consequences. The drawback of this design is that two of the pinpointed SNPs in the replication analysis, rs13140552 and rs4891564, were only carried by five controls and two cases, respectively. Therefore, even if the Chi-square test resulted in significant associations, it would be adventurous to draw clinical conclusions from this specific finding. In addition, the validation study would have benefited from a larger sample size, which could in part have made up for the low allele frequencies analyzed. Another limitation is that the DKD diagnosis was not confirmed by biopsy in all patients, which precluded expression studies from being performed. These could have been interesting to unveil underlying mechanisms for the genotype–phenotype associations reported, especially when three of the four relevant genes identified (*MHY9*, *RGMA* and *SOWAHB* are highly expressed in the human kidney (www.proteinatlas.org, accessed on 1 July 2021). Finally, functional studies that could shed some light on the mechanisms explaining the SNP effects are generally lacking, and the in silico tools that were utilized in the present work could not identify clear functional consequences for the studied variants. The present work adds to the considerable efforts that are currently being made to identify genetic biomarkers of DKD, efforts that are very much needed because there remains considerable unexplained heritability in this disease [33]. Very recently, Lazaro-Guevara et al. [34] analyzed 345 kidney disease related genes by NGS in 206 diabetic and non-diabetic renal patients. The authors did not include regulatory regions in the analysis and there was no replication study, but they found that roughly one-fifth of DKD patients carried rare variants that could contribute to their disease.

## 5. Conclusions

By utilizing a custom target NGS 70-gene panel and a validation cohort, we have been able to point out some of these rare variants in the *MYH9*, *CNDP1*, *SOWAHB* and *RGMA* genes relevant to renal disease in diabetic patients. Further studies on larger and more diverse replication cohorts, as well as expression studies and/or validation in experimental DKD models, are warranted to confirm the results reported herein.

## Figures and Tables

**Table 1:** Demographic and clinical characteristics of the discovery cohort. Data are shown as median (interquartile range), mean ± standard deviation or count (percentages).

	Controls	DKD	DKD-ESRD
N	50	50	50
Age (years)	70 (7)	69 (13)	70 (16)
Sex			
Women	16 (32.0)	15 (30.0)	13 (26.0)
Men	34 (68.0)	35 (70.0)	37 (74.0)
Weight (kg)	78 (18.45)	82.5 (20.25)	72.5 (23.5)
Proteinuria (mg/24 h)	92.4 (135.3)	390 (888.71)	
Albuminuria (mg/24 h)			
<30	28 (59.6)	10 (21.7)	
30–300	13 (27.7)	18 (39.1)	
>300	6 (12.8)	18 (391)	
Serum creatinine (mg/mL)	0.72 (0.38)	0.61 (0.30)	
Albumin/Creatinine (mg/g)	13.16 (80.96)	168.17 (594.99)	
Creatinine clearance (mL/min)	89.42 (67.97)	57.18 (38.60)	
eGFR (mL/min)	69.2 (62.5)	43.7 (33.7)	
Glucose (mg/dL)	101 (13)	137 (61)	178 (123)
HbA1c (%)	6 (1.3)	7.1 (1.3)	7.6 (1.5)
Smoking			
No	31(62.0)	16 (32.0)	24 (48.0)
Yes (including former smokers)	19 (38.0)	34 (68.0)	26 (52.0)
Systolic blood pressure (mmHg)	133.0 ± 28.9	144.1 ± 22	140.1 ± 28
Diastolic blood pressure (mmHg)	70.9 ± 11.2	79.0 ± 11	69 ± 12.1
Pulse pressure (mmHg)	62.1 ± 22.2	65.0 ± 19.1	71.1 ± 19.9
Hypertension			
No	11 (22.0)	12 (24.0)	2 (4.0)
Yes	39 (78.0)	38 (76.0)	48 (96.0)
Hyperlipidemia			
No	35 (70.0)	36 (72.0)	21 (42.0)
Yes	15 (30.0)	14 (28.0)	29 (58.0)
Evolution of DM (years) ^a^			
0–10	-	11 (22.4)	4 (8.2)
10–20	-	21 (42.9)	22 (44.9)
>20	-	17 (34.7)	23 (46.9)

DKD, diabetic kidney disease; ESRD, end-stage renal disease; eGFR, estimated glomerular filtration rate; Hb1Ac, glycosylated hemoglobin. ^a^ Data were missing for two subjects.

**Table 2:** List of gene variants found to be relevant in the discovery cohort and that were subsequently tested in the replication cohort. Rs number is shown when available.

Chromosome	Position	Gene	Reference Allele	Alternate Allele	rs	Highest Population MAF	OR	CI	*p*-Value
Controls vs. DKD groups									
11	77300435	*AQP11*	T	A			4.33	(2.21–8.57)	3.82 × 10^−6^
1	22206942	*HSPG2*	G	G	rs1874793	0.035	0.05	(0.007–0.4)	1.5 × 10^−4^
4	103538411	*NFKB1*	T	A			0.06	(0.007–0.44)	3.1 × 10^−4^
1	230841687	*AGT*	T	C	rs7080	0.14	0.17	(0.05–0.51)	7.7 × 10^−4^
18	72252086	*CNDP1*	A	C	rs4891564	0.06	0.11	(0.02–0.48)	7.9 × 10^−4^
1	186946912	*PLA2G4A*	G	A	rs2307198	0.05	0.11	(0.01–0.48)	7.9 × 10^−4^
4	103538177	*NFKB1*	A	T			13.5	(1.72–105.93)	0.002
16	24230479	*PRKCB*	A	T			0.07	(0.009–0.58)	0.002
9	130889841	*PTGES2*	A	T			0.16	(0.04–0.57)	0.003
4	175598334	*GLRA3*	T	C	rs6812439	0.12	0.13	(0.03–0.57)	0.003
9	130883511	*PTGES2*	C	T	rs2040004	0.33	0.13	(0.03–0.57)	0.003
9	130890281	*PTGES2*	C	G	rs6478820	0.16	0.13	(0.03–0.57)	0.003
22	36691607	*MYH9*	A	C	rs710181	0.06	0.22	(0.08–0.63)	0.004
11	102713447	*MMP3*	G	C	rs41380244	0.14	12.23	(1.54–96.68)	0.005
15	93587438	*RGMA*	A	G	rs3752103	0.49	0.18	(0.05–0.62)	0.005
7	29394249	*CHN2*	G	T			7.25	(1.59–33.02)	0.005
18	72188371	*CNDP2*	A	G	rs890334	0.22	0.19	(0.05–0.68)	0.009
2	174820817	*SP3*	A	G			5.26	(1.46–18.94)	0.009
4	77818132	*SOWAHB*	G	C	rs13140552	0.16	11.00	(1.38–87.64)	0.009
4	175565010	GLRA3	A	C			9.79	(1.22–78.81)	0.018
15	93588336	*RGMA*	A	C	rs4238485		3.62	(1.27–10.3)	0.019
7	29513367	*CHN2*	T	C	rs1059185	0.49	2.15	(1.14–4.08)	0.04
DKD vs. DKD with ESRD groups									
11	102795585	*MMP1*	T	C	rs470558	0.21	0.15	(0.03–0.69)	0.01
15	93044800	*RGMA*	T	C	rs1969589	0.48	0.08	(0.01–0.64)	0.005
Renal function and damage *									
1	186672494	*PTGS2*	A	C	rs2853805		5.53	(1.55–9.2)	0.019
9	35161846	*UNC13B*	T	G			11.00	(1.23–83.40)	0.004
15	94946287	*MCTP2*	C	A	rs16949097	0.22	8.70	(1.33–7.45)	0.013
15	93588309	*RGMA*	C	G	rs62021480	0.27	5.91	(1.13–25.57)	0.026

DKD, diabetic kidney disease; ESRD, end-stage renal disease; MAF, minor allele frequency; OR, odds ratio; CI, 95% confidence intervals. * As estimated by glomerular filtration rate and proteinuria (albumin-to-creatinine ratio) values.

**Table 3:** Demographic and clinical characteristics of the replication cohort. Data are shown as median (interquartile range) or count (percentages).

	Controls	DKD
N	506	318
Age (years)	57 (17)	63 (18)
Sex		
Women	232 (45.8)	110 (34.6)
Men	274 (54.2)	208 (65.4)
Weight (kg)	76 (20.3)	78.3 (20.3)
HbA1c (%)	5.6 (0.9)	7.1 (1.8)
Proteinuria (mg/24 h)	95.1 (140.1)	1612 (2872.5)
Glucose (mg/dL)	102 (20)	139.5 (80)
Albumin/Creatinine (mg/g)	7.2(45.1)	186.4 (841.6)
eGFR (mL/min)	89.2 (21.8)	29.4 (21.2)
Smoking		
No	195 (38.5)	123 (38.7)
Yes (including former smokers)	311 (61.5)	195 (61.3)
Systolic blood pressure (mmHg)	133.7 ± 17.8	144.9 ± 24.0
Diastolic blood pressure (mmHg)	80.04 ± 9.7	79.2 ± 11.8
Pulse pressure (mmHg)	53.6 ± 13.2	71.5 ± 20.1
Hypertension		
No	325 (64.2)	4 (1.3)
Yes	181 (35.8)	314 (98.7)
Hyperlipidemia		
No	327 (64.6)	58 (18.2)
Yes	179 (35.4)	260 (81.8)

DKD, diabetic kidney disease; eGFR, estimated glomerular filtration rate.

**Table 4:** Adjusted risk analysis of replicated genetic variants with diabetic kidney disease.

SNP	Gene	Genotype	Control	%	DKD	%	OR	CI	*p*-Value
rs13140552	*SOWAHB*	G/G-C/G	498	99.0	318	100.0	Ref.		0.044
		C/C	5	1.0	0	0	-		
rs4891564	*CNDP1*	C/C	504	100.0	316	99.4	Ref.		0.023
		A/C	0	0	2	0.6	-		
rs710181	*MYH9*	C/C	464	91.9	303	95.3	Ref.		0.033
		A/C-A/A	41	8.1	15	4.7	0.52	(0.28–0.97)	

DKD, diabetic kidney disease; OR, odds ratio; CI, 95% confidence intervals.

## Data Availability

The datasets underlying this article can be found at the Figshare repository with doi:10.6084/m9.figshare.16685296.

## Supplementary Materials

The following are available online at https://www.mdpi.com/article/10.3390/genes12121992/s1. Figure S1: Number of genetic variants detected in each of the main biological processes in which the 70 candidate genes were involved distributed in the three study cohorts. Table S1: List of genetic variants whose frequencies were significantly different between controls and diabetic kidney disease patients in the discovery cohort. Rs numbers are provided when available.

## Abbreviations

BWA	Burrows–Wheeler Aligner
CKD	Chronic kidney disease
DKD	Diabetic kidney disease
eGFR	Estimated glomerular filtration rate
ESRD	End-stage renal disease
GO	Gene Ontology
NGS	Next-generation sequencing
OMIM	Online Mendelian Inheritance in Man
OR	Odds ratio
T2D	Type 2 diabetes
