# Supplementary material for: A Custom Target Next-Generation Sequencing 70-Gene Panel and Replication Study to Identify Genetic Markers of Diabetic Kidney Disease
Source: Genes (Basel). 2021 Dec 15;12(12):1992. doi: 10.3390/genes12121992 (PMC8702126; doi:10.3390/genes12121992)
Supplement: Supplementary file 1 [file genes-12-01992-s001.zip › Supplementary Tables.pdf]

genes

SUPPLEMENTARY TABLE S1. List of genetic variants whose frequencies were significantly different between controls and diabetic kidney disease patients in the discovery cohort. Rs numbers are provided when available.

Chromosome	Position	Gene	Reference allele	Alternate allele	rs number	p-value
1	22206942	*HSPG2*	G	G	rs1874793	0.000154
9	125156982	*PTGS1*	T	A		0.000198
4	103538411	*NFKB1*	T	A		0.000315
12	48298867	*VDR*	C	G		0.000525
1	230841687	*AGT*	C	T	rs7080	0.000769
18	72252086	*CNDP1*	T	A	rs4891564	0.000791
1	186946912	*PLA2G4A*	A	G	rs2307198	0.000791
7	151253058	*PRKAG2*	T	A		0.000829
9	130890060	*PTGES2*	T	A		0.001089
4	103538177	*NFKB1*	A	T		0.002539
16	24230479	*PRKCB*	A	T		0.002539
9	130889841	*PTGES2*	A	T		0.002846
4	175598334	*GLRA3*	C	T	rs6812439	0.002902
9	130883511	*PTGES2*	T	C	rs2040004	0.002902
7	36893232	*ELMO1*	T	A	rs7804092	0.003325
22	36691607	*MYH9*	C	A	rs710181	0.003872
7	29552463	*CHN2*	G	C	rs1420145	0.004242
15	94946231	*MCTP2*	A	C	rs11633973	0.004392
11	102713447	*MMP3*	G	C	rs41380244	0.004982
15	93587438	*RGMA*	G	A	rs3752103	0.005192
9	125133479	*PTGS1*	T	C	rs1236913	0.005427
7	29394249	*CHN2*	G	G		0.005451
18	72188371	*CNDP2*	A	G	rs890334	0.009329
7	150711671	*NOS3*	A	C		0.009329
2	174820817	*SP3*	A	G		0.009329
4	77818132	*SOWAHB*	G	C	rs13140552	0.009658
19	15989040	*CYP4F2*	C	G	rs1272	0.014529
4	175565010	*GLRA3*	A	C		0.018488
15	93588336	*RGMA*	C	A	rs4238485	0.019243
22	36662626	*APOL1*	A	G		0.021434
19	45409167	*APOE*	C	C	rs440446	0.023068
7	29552983	*CHN2*	T	C	rs1059185	0.026118
16	20360101	*UMOD*	G	G	rs7193058	0.027827

19	16006413	*CYP4F2*	G	A	rs3093114	0.027943
19	16008388	*CYP4F2*	A	C	rs3093105	0.027943
12	48236550	*VDR*	G	C	rs2544043	0.028671
3	148459395	*AGTR1*	C	T	rs5182	0.030752
1	71471781	*PTGER3*	G	A	rs2744915	0.031101
1	71477315	*PTGER3*	A	T	rs5680	0.031101
7	150696111	*NOS3*	T	G	rs1799983	0.031882
16	55523705	*MMP2*	T	C	rs243849	0.035530
1	230838258	*AGT*	G	A	rs2067853	0.035568
1	230838331	*AGT*	G	T	rs7079	0.035568
1	22165901	*HSPG2*	A	G	rs2291826	0.038099
15	95023525	*MCTP2*	G	G	rs1047084	0.044932
9	85862365	*FRMD3*	G	A	rs1056476	0.047165
18	72238472	*CNDP1*	C	A	rs73973908	0.048402
12	48236386	*VDR*	T	A	rs2853562	0.048918

SUPPLEMENTARY TABLE S2. List of genetic variants whose frequencies were significantly different between diabetic kidney disease patients with and without ESRD in the discovery cohort. Rs numbers are provided when available.

Chromosome	Position	Gene	Reference allele	Alternate allele	rs number	p-value
12	48236550	*VDR*	G	C	rs2544043	3.50E-08
12	48236386	*VDR*	T	A	rs2853562	0.0004
11	77300435	*AQP11*	T	T		0.0005
4	103538411	*NFKB1*	T	A		0.0013
2	100210478	*AFF3*	A	G		0.0015
15	94841430	*MCTP2*	G	G		0.0022
15	94841691	*MCTP2*	G	A	rs61737195	0.0022
7	150711701	*ATG9B;NOS3*	G	T	rs866566532	0.0025
4	103538177	*NFKB1*	A	T		0.0025
15	93588030	*RGMA*	T	T		0.0050
15	93588336	*RGMA*	A	A		0.0081
11	102666316	*MMP1*	T	T		0.0101
2	100218080	*AFF3*	G	A	rs4851214	0.0138

7	151253765	*PRKAG2*	C	C		0.0154
7	29552855	*CHN2*	C	T	rs76079432	0.0192
7	36895342	*ELMO1*	C	T	rs13246439	0.0193
7	151253083	*PRKAG2*	C	C		0.0226
7	151253226	*PRKAG2*	C	C		0.0226
7	151254175	*PRKAG2*	T	T		0.0226
7	150711671	*NOS3*	A	C		0.0238
14	102698131	*MOK*	G	A	rs7152550	0.0249
14	102729881	*MOK*	A	G	rs56377169	0.0249
19	45409131	*APOE*	C	C		0.0309
7	151573731	*PRKAG2*	G	A	rs66628686	0.0309
16	24230479	*PRKCB*	A	T		0.0349
14	52781381	*PTGER2*	A	G		0.0349
4	77816125	*SOWAHB*	A	G		0.0349
7	29552983	*CHN2*	T	C	rs1059185	0.0401
5	132009614	*IL4*	T	T		0.0414
1	230849872	*AGT*	C	T	rs5051	0.0423
18	72234635	*CNDP1*	C	C		0.0461
3	148460700	*AGTR1*	G	A	rs380400	0.0464
19	45409296	*APOE*	T	T		0.0489
15	33025979	*GREM1*	C	C		0.0494
