# Supplementary figures and images for: A Custom Target Next-Generation Sequencing 70-Gene Panel and Replication Study to Identify Genetic Markers of Diabetic Kidney Disease
Source: Genes (Basel). 2021 Dec 15;12(12):1992. doi: 10.3390/genes12121992 (PMC8702126; doi:10.3390/genes12121992)

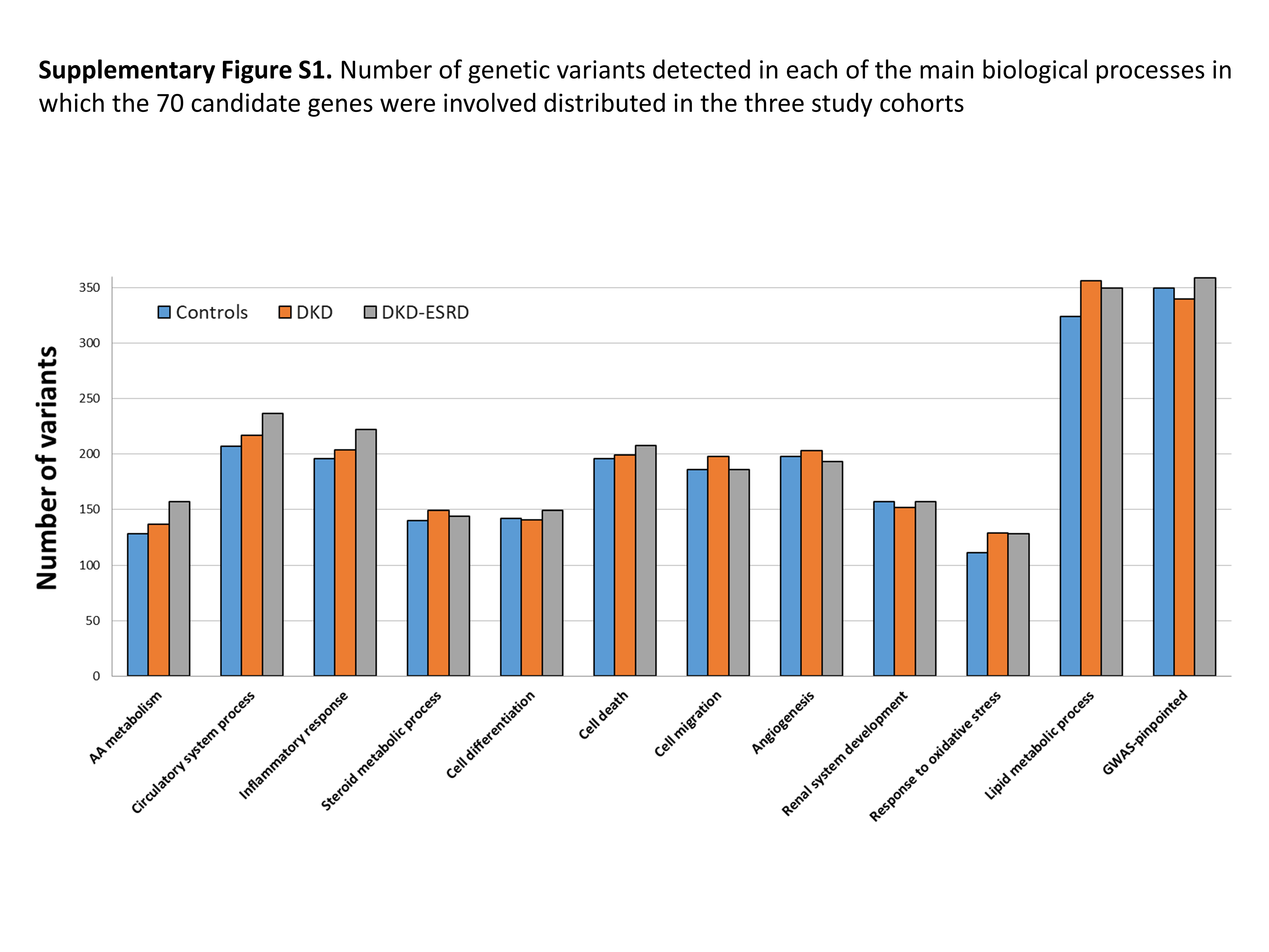

Supplement: Supplementary file 1 [file genes-12-01992-s001.zip › Suppl Figure S1.TIF]
